# Complete Genome Sequence of Stenotrophomonas maltophilia Siphophage Siara

**DOI:** 10.1128/mra.00177-22

**Published:** 2022-05-02

**Authors:** John Marmion, Nathaniel Tate, James Clark, Tram Le, Ben Burrowes, Mei Liu

**Affiliations:** a Department of Biochemistry and Biophysics, Texas A&M University, College Station, Texas, USA; b Center for Phage Technology, Texas A&M University, College Station, Texas, USA; c BB Phage Consultancy, LLC, Georgetown, Texas, USA; Loyola University Chicago

## Abstract

Stenotrophomonas maltophilia is associated with an increasing incidence of nosocomial infections. Here, we describe the isolation and genome annotation of S. maltophilia siphophage Siara. Its 61,427-bp genome is currently related only to one phage in the NCBI database, namely, S. maltophilia phage Salva, and is not related to any prophages.

## ANNOUNCEMENT

The increasing incidence of nosocomial infection caused by Stenotrophomonas maltophilia is concerning due to some strains being multidrug resistant and the significant infection fatality-to-case ratio (1). The isolation and genome annotation of phage Siara, a potential therapeutic agent for controlling multidrug-resistant S. maltophilia are described here.

Phage Siara was isolated in 2019 from an influent water sample collected from a wastewater treatment plant in Beaumont, TX (Global Positioning System [GPS] coordinates of 30.20078, −94.10807), using S. maltophilia (ATCC 17807) as the host. The host strain was propagated aerobically at 30°C in tryptone nutrient (0.5% tryptone, 0.25% yeast extract, 0.1% glucose, and 0.85% NaC [wt/vol]) broth or agar. Phage isolation and propagation were done using the soft agar overlay method (2). Phage DNA was purified from polyethylene glycol (PEG)-precipitated phage particles from ~8 mL phage lysate (>10^9^ PFU/mL) using a Promega Wizard DNA cleanup system following the manufacturer’s protocol (3). The purified DNA was prepared as 300-bp inserts using a Swift 2S Turbo library preparation kit and sequenced on an Illumina MiSeq instrument with paired-end 150-bp reads using V2 300-cycle chemistry. A total of 174,160 raw sequence reads were quality controlled with FastQC (http://www.bioinformatics.babraham.ac.uk/projects/fastqc) and trimmed using the FASTX-Toolkit v0.0.14 (http://hannonlab.cshl.edu/fastx_toolkit/) to generate a total of 125,775 trimmed reads, from which a single contig with 129-fold coverage was assembled with SPAdes v3.5.0 (4). Contig end sequences were verified by PCR and Sanger sequencing the resulting product using primers 5′-TGCTGCCGTTCACAAAACAG-3′ and 5′-TCCTGACTCTACCCACCCTG-3′. The phage termini were predicted using PhageTerm (5). The structural annotation was done using GLIMMER v3 (6) and MetaGeneAnnotator v1.0 (7), and tRNAs were detected with ARAGORN v2.38 (8) and tRNAScan-SE v2.0 (9). The gene functions were predicted using InterProScan v5.48 (10), TMHMM v2.0 (11), HHpred (12), LipoP v1.0 (13), and SignalP v5.0 (14), as well as BLAST (15) searches against the following databases: NCBI nonredundant and SwissProt (16). Rho-independent termination sites were annotated using TransTermHP v2.09 (17). progressiveMauve v2.4 (18) was used to calculate the genome-wide similarity. Analysis tools were accessed through the Center for Phage Technology (CPT) Galaxy-Apollo platform (https://cpt.tamu.edu/galaxy-pub) (19–21), and all software were used at their default settings. The morphology of phage Siara was determined by transmission electron microscopy (TEM) and viewing samples stained negatively with 2% (wt/vol) uranyl acetate at the Texas A&M Microscopy and Imaging Center.

**FIG 1:**
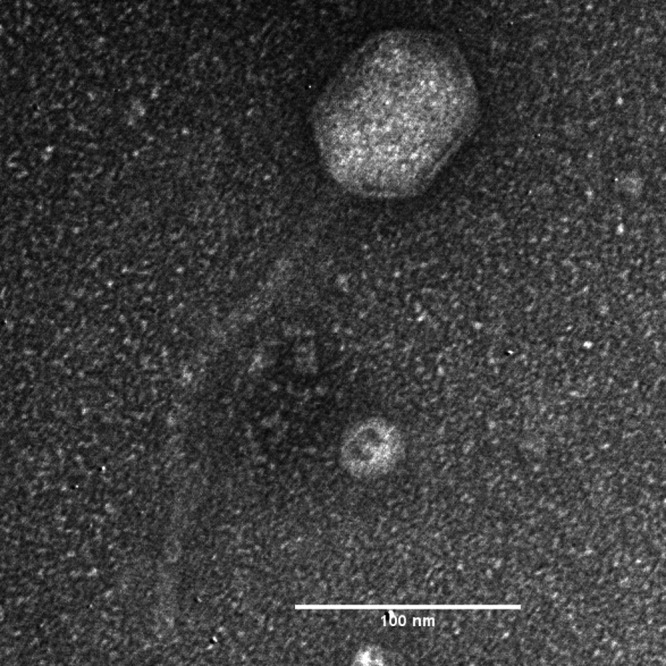
Transmission electron micrograph of phage Siara. Phage particles were diluted with TEM buffer (20 mM NaCl, 10 mM Tris-HCl [pH 7.5], and 2 mM MgSO_4_) and captured on a freshly glow-discharged, Formvar carbon-coated grid. The grids were stained with 2% (wt/vol) uranyl acetate and observed on a JEOL 1200 EX transmission electron microscope at a 100-kV accelerating voltage at the Microscopy and Imaging Center at Texas A&M University.

Phage Siara is determined to have a siphophage morphology (Fig. 1). Siara has a complete genome length of 61,427 bp, a coding density of 93.9%, and a G+C content of 56.5%. A total of 100 protein-coding genes and 3 tRNA genes were identified in the Siara genome. At the time of writing of this manuscript, phage Siara is closely related to only one phage deposited in GenBank, namely, phage Salva (GenBank accession number MW393850) (22), sharing 86.7% nucleotide identity over 73% Siara genome coverage as determined by BLASTn. Similar to the long terminal repeat region (3,973 bp) identified in phage Salva, PhageTerm analysis identified a putative 2,528-bp terminal repeat region in the Siara genome. Phage Siara and Salva are currently unclassified members within the *Siphoviridae* family. Based on BLASTn at an overall nucleotide similarity cut off 30% (calculated by using the percent identity times the percent aligned length, as determined by BLASTn), Siara is not closely related to any prophage elements in NCBI bacterial genomes.

### Data availability.

Siara was deposited in GenBank with accession number MZ326859. The associated BioProject, SRA, and BioSample accession numbers are PRJNA222858, SRR14095253, and SAMN18509351, respectively.
